# Novel variants ensued genomic imprinting in familial central precocious puberty

**DOI:** 10.1007/s40618-023-02300-3

**Published:** 2024-02-17

**Authors:** V. Karaman, E. Karakilic-Ozturan, S. Poyrazoglu, M. Y. Gelmez, F. Bas, F. Darendeliler, Z. O. Uyguner

**Affiliations:** 1 Department of Medical Genetics, Istanbul Faculty of Medicine, Istanbul University, Millet Cad. Çapa/Fatih, 34096 Istanbul, Turkey; 2 Department of Pediatric Endocrinology and Diabetes, Istanbul Faculty of Medicine, Istanbul University, Istanbul, Turkey; 3 Department of Immunology, Aziz Sancar Institute of Experimental Medicine, Istanbul University, Istanbul, Turkey

**Keywords:** Central precocious puberty, *DLK1*, *MKRN3*

## Abstract

**Introduction:**

Central precocious puberty (CPP) is characterized by the early onset of puberty and is associated with the critical processes involved in the pubertal switch. The puberty-related gene pool in the human genome is considerably large though few have been described in CPP. Within those genes, the genomic imprinting features of the *MKRN3* and *DLK1* genes add additional complexity to the understanding of the pathologic pathways. This study aimed to investigate the molecular etiology in the CPP cohort.

**Methods:**

Eighteen familial CPP cases were investigated by Sanger sequencing for five CPP-related genes; *DLK1*, *KISS1*, *KISS1R*, *MKRN3*, and *PROKR2*. Segregation analysis was performed in all patients with pathogenic variants. Using an ELISA test, the functional pathogenicity of novel variants was also investigated in conjunction with serum delta-like 1 homolog (DLK1) concentrations.

**Results:**

In three probands**,** a known variant in the *MKRN3* gene (c.982C>T/p.(Arg328Cys)) and two novel variants in the *DLK1* gene (c.357C>G/p.(Tyr119Ter) and c.67+78C>T) were identified. All three were inherited from the paternal allele. The individuals carrying the *DLK1* variants had low detectable DLK1 levels in their serum.

**Conclusions:**

The frequencies were 5.5% (1/18) for *MKRN3* 11% (2/18) for *DLK1*, and none for either *KISS1*, *KISS1R*, and *PROKR2*. Low serum DLK1 levels in affected individuals supported the relationship between here described novel *DLK1* gene variants with CPP. Nonsense nature of c.357C>G/p.(Tyr119Ter) and an alteration in the evolutionarily conserved nucleotide c.67+78C>T suggested the disruptive nature of the variant's compatibility with CPP.

## Introduction

Central precocious puberty (CPP) occurs due to early reactivation of the hypothalamic–pituitary–gonadal (HPG) axis leading to the development of secondary sexual characteristics before the age of 8 in girls and 9 in boys [1–3]. The onset of puberty is a complex process influenced by various factors, including hormonal, genetic, environmental, ethnic, nutritional, and socio-economic factors. The effect of genetic factors is estimated to be around 50–80%. It has been determined that approximately 27.5% of CPP cases show familial transition with autosomal dominant inheritance. It is expected more frequently in females than in males, and it is estimated that 90% of females and more than 25% of males with CPP are idiopathic [2, 4, 5]. Although more than 70 genes have been reported today in puberty, the ones mainly associated with idiopathic or familial CPP are the gain-of-function mutations in *KISS1*, *KISS1R*, and *PROKR2*, and the loss-of-function variant in paternally inherited allele in the *MKRN3* and the *DLK1* genes [1, 6–10]. The earliest CPP association was for the *KISS1R* gene variant detected in an adopted girl with CPP in 2008, which was shown to increase the intracellular signal duration after the binding of kisspeptin, which stimulates the secretion of GnRH in the hypothalamus [7]. Later in 2010, the involvement of *KISS1* was revealed in a male child with idiopathic CPP detected with an increased testicular volume at the age of one. The situation is attributed to the variant with a gain-of-function role leading to the increased stability of KISS1 in the hypothalamic KISS1/KISS1R system, which has a central regulatory character in the gonadotropic axis at puberty [8]*.* In 2017, a monoallelic frame-shift deletion variant in the *PROKR2* gene was revealed in 3,5 years old girl with CPP and her mother with normal-age puberty [9]. Although the variant is a truncation type, co-expressing cells with wild and mutant types lead to an increase of ligand-induced Ca^2+^ level hence elongation of the signal induction activity, thereof attributed to gain-of-function [6, 9, 11]. Based on this single report, the association of *PROKR2* with CPP is currently under suspicion. The association of the *MKRN3* and the *DLK1* gene is intriguing in that both exhibit genomic imprinting properties. Paternally expressed *MKRN3* is essential in regulating puberty's timing by inhibiting the pubertal pulsatile GnRH secretion and upstream suppressor of the HPG axis [10, 12, 13]. Unlike *KISS1*, *KISS1R* and *PROKR2*, loss-of-function type alterations in the paternal allele in the *MKRN3* gene account for CPP via disposal of its inhibitory role in the onset of puberty [9, 12, 14, 15]. The role of DLK1, which consists of six epidermal growth factor (EGF) repeat-containing transmembrane domains, in puberty's timing is unclear. However, there is a suggestion that the activation or inhibition of the Notch target genes in the hypothalamus indirectly regulates the formation, maturation, and secretion of kisspeptin neurons. This establishes a link between *KISS1* and *MKRN3* [16, 17]. *DLK1* also illustrates its function through the paternally expressed allele via the kisspeptin signaling pathway in managing pubertal timing neurogenesis and adipose tissue homeostasis [10, 13]. The *MKRN3* gene is currently the most commonly implicated in familial central precocious puberty. However, studies have also suggested a potential role for the *DLK1* gene in CPP. Loss-of-function mutations in *DLK1* have been found in a small number of individuals with familial CPP, leading to early-onset puberty. These alterations affect the function of DLK1 and contribute to the development of precocious puberty [10, 16, 17]. This study aimed to examine possible underlying genetic causes in patients with familial CPP.

## Materials and methods

### Patients

Eighteen patients with familial CPP from unrelated Turkish descents were enrolled in the study. The familial form of CPP was defined by the presence of at least one case among relatives (first or second degree). Some clinical and laboratory findings were recorded retrospectively from patient files. A detailed family history was obtained and examinations were conducted for all patients during the study. Documented CPP, menarche age (≤ age of 10 years) for female relatives, and age of any pubertal signs (such as early voice breaking, facial shaving, and growth spurt) for male relatives were taken into consideration[18].

### Methods

The diagnosis of CPP based on breast Tanner stage II and testicular volume of 4 ml before the age of 8 years in girls and 9 years in boys, respectively, along with basal (> 0.6 mIU/ml) and/or stimulated (> 5 mIU/ml) LH levels. Pubertal development was assessed using the Tanner-Marshall scale [19, 20]. Height and weight were measured in all subjects and their parents using a wall-mounted calibrated Harpenden Stadiometer (Holstein Ltd, Crymych, United Kingdom) sensitive to 0.1 cm and an electronic scale sensitive to 0.1 kg. Body mass index (BMI) was determined as weight (kg)/height (m^2^). All measurements were expressed as standard deviation scores (SDS) according to age and birth-registered sex appropriate to national standards [21]. Bone age (BA) was evaluated by Greuclich-Pyle-method and predicted adult height (PAH) was calculated according to Bayley-Pinneau-method. Luteinizing hormone (LH) and follicle-stimulating hormone (FSH) were analyzed by ECLIA (Cobas, Roche Diagnostics, Mannheim, Germany). Testosterone (T), and Estradiol (E2) levels were studied by immune-chemiluminescence assay (ICMA) (Immulite 2000 system, Siemens AG, Berlin and Munich, Germany). The pubertal suppression was initiated with GnRH analog (a) at standard doses (3.75 mg/4 weeks or 11.25 mg/12 weeks). Cranial MRI was performed in all cases to rule out organic pathologies. Pelvic ultrasound (US) was conducted to evaluate the dimension of the uterus and ovaries in girls. The local Ethics Committee of Istanbul University Istanbul Faculty of Medicine approved the study (reference number 14.08.2018/1124).

### Genetic investigations

Written informed consent was obtained from all patients, their parents, and related family members previous to the genetic investigation. Pedigrees were drawn, and 2 ml venous blood samples were collected for genomic DNA isolations (MagPurix Kit 200; Zinexts Life Science Corp. Medsantek, Istanbul) in accordance with the manufacturer’s instructions. Ensembl was used for human reference gene sequences to design the primers, originality was tested at UCSC in silico PCR program. Polymerase chain reaction (PCR) amplified in thermal cyclers (SimpliAmp, Applied biosystems, and DNA Engine T100; BIORAD) and subsequently Sanger sequencing (SS) of the *KISS1R* (NM_032551.5, 5 exons), *KISS1* (NM_002256.4, 3 exons), *PROKR2* (NM_144773.4, 2 exons), *MKRN3* (NM_005664.3, 1 exon) and *DLK1* (NM_003836.7, 5 exons) genes were performed (ABI 3500, Applied Biosystems). Detected variants are searched by using literature, dbSNP[22] and GnomAD[23], Human Genome Mutation Database (HGMD) [6] and ClinVar [24]. Pathogenicity assessments are made by using mutation taster [25], Polyphen2 [26], Splice AI [27]and the classification of the variants according to ACMG based on VarSome [28], and Franklin databases (https://franklin.genoox.com/). Segregation studies were conducted in families in the presence of the identified variant [25, 26]. The MKRN3 and DLK1 peptide domains were arranged as indicated in the Uniprot (a worldwide hub of protein knowledge) database [29].

### Biochemical functional study (serum DLK1 measurements)

Soluble DLK1 ELISA (enzyme-linked Immunosorbent assay) test kit is used following the manufacturer’s instructions Bioassay Technology Laboratory (BT LAB—Lot: 202109018—Cat# E5959Hu, RRID:AB_2941902 Zhejiang, China). Serum samples were collected and immediately frozen on the same day and then stored at − 80 °C for preservation. Serum DLK1 levels are measured for individuals and their family members (*n* = 11). The control group (*n* = 54) for DLK1 level was composed of prepubertal, healthy girls with no significant chronic diseases. The intra- and interassay variations were < 8% and < 10, respectively. The assay has a range of 20–6000 ng/L and a sensitivity of 9.47 ng/L.

### Statistical analysis

Comparisons between serum DLK1 concentration of controls and affected individuals with CPP carrying paternally inherited *DLK1* gene variants were performed using an unpaired *t*-test and ordinary one-way ANOVA using GraphPad Prism 9.5.0. (GraphPad Software, San Diego, CA).

## Results

### Clinical findings

The CPP cohort is composed of 18 patients (CPP 1–18); 16 females (%88.8), and 2 males (%11.2; CPP-7 and CPP-8). The clinical and laboratory findings and follow-up of all patients are detailed in Table 1. All values for females are given in mean (Interquartile range; IQR) and independently disclosed for males since only two males were in the study group. The age of the onset of puberty was 7.3 (0.5) years in females; 6.7 and 8.8 years in males. All cases were born at term and the median birth weight-SDS was − 0.65 (0.42) in females and − 0.1 in males. At the onset of puberty, the median height and BMI-SDS were − 0.4 (1.9) and 1.1 (1.2) in females, 2.4 (0.3) and 1.7 in males, respectively. The median target height SDS was − 0.7 (0.9) in females; − 0.68 and 0.32 in males. The median BA was 8.83 (1.2) years, and the chronological age-BA difference was − 1.33 (0.53). The median PAH was 154.8 cm (− 1.4 SDS) in females; 167 and 181.7 cm (− 1.5 and 0.9 SDS) in males. All patients had normal cranial-pituitary magnetic resonance imaging (MRI) findings and pelvic ultrasonography supported the onset of puberty in girls. Fifteen cases received GnRHa treatment. The initial age of treatment was 8.9 (0.6) years in females; 6.8 and 9.7 years in males. GnRHa treatment was discontinued at the age of 11.2 (0.9) years in females; 10.2 and 12.8 years in males. An escalation in the treatment dosage (7.5 mg/month) was required in two cases (CPP-5 and CPP-11). There was no short stature in nine patients (2, 4, 7, 8, 12–16 and 18) who were clinically followed up regularly, received treatment, and were known to have reached the final height (Table 1).

**Table 1:** Clinical and hormonal findings of patients with familial central precocious puberty

Patients	**Sex**	Cons	At diagnosis													The age of the start of GnRH treatment (yrs)	The duration of GnRH treatment (yrs)	Final Height cm (SDS)	ΔFinal Height-target height (SDS)
			The age of puberty onset (yrs) #	The age at the referral time (yrs)#	Height cm (SDS)	BMI kg/m^2^ (SDS)	Puberty tanner	Bone Age (yrs)	PAH cm (SDS)	Target height cm (SDS)	LH (IU/L)	FSH (IU/L)	E2 (pg/mL) /T (ng/mL)	Stimulated LH (IU/L)	Stimulated FSH (IU/L)				
1	F	–	7.5	9.2	134.5 (0.2)	25.3 (2.4)	2	10	156 (− 1.2)	161 (− 0.4)	0.1	2.7	6.7 / −	5.2	16.6	−	−	NA	−
2	F	−	6.8	6.8	117.8 (− 0.4)	16.2 (0.3)	2	NA	NA	162.5 (− 0.1)	0.1	1.3	19.1 / −	5.3	9.1	9.6	3	164.5 (0.2)	+ 0.3
3*	F	−	7	7.4	136.3 (2.5)	19.2 (1.4)	4	8.83	166 (0.5)	160 (− 0.5)	0,6	1.4	15 / −	3.3	12.8	7.4	3.6	167.4 (0.84)	+ 1.34
4**	F	−	7	8.4	138.9 (1.8)	16.3 (0.1)	4	10.5	155.5 (− 1.3)	162.3 (− 0.1)	1.6	6.6	76 / −	−	−	8.7	2.1	156.8 (− 1.1)	− 1.0
5	F	1° cousins	7.5	9.0	134 (0.3)	17.5 (0.5)	3	10.5	151.5 (− 2.0)	161.6 (− 0.3)	2.7	6.9	83.1 / −	−	−	9.2	1.6	NA	−
6	F	−	7	7.5	134.9 (1.9)	21.4 (1.9)	3	7.83	171.6 (1.5)	158.4 (− 0.8)	2.6	1	9.5 / −	−	−	9.8	1.2	NA	−
7	M	−	8.8	8.8	142.5 (0.5)	21.4 (0.9)	2	10	181.7 (0.9)	178.8 (0.4)	2.3	0.9	− / 0.3	−	−	9.7	0.5^+^	180 (0.6)	+ 0.2
8	M	−	6.7	6.7	132.8 (2.6)	25 (3.2)	2	10.5	167 (− 1.5)	171.5 (− 0.8)	0.1	1.6	− /0.2	5.5	5.2	6.8	6.0	178.5 (0.3)	+ 1.1
9	F	1° cousins	7.5	8.8	132.9 (0.3)	17.1 (0.4)	2	8.83	161.9 (− 0.2)	160.9 (− 0.4)	0.8	4	40.4 / −	−	−	−	−	NA	−
10	F	−	7.5	8.4	129.6 (0.2)	16.1 (0.03)	2	8.83	157.8 (− 0.9)	148.9 (− 2.4)	0.7	4	47.4 / −	6.2	10.8	8.9	2.1	NA	−
11	F	−	6.4	6.4	114.7 (− 0.5)	15.1 (− 1.1)	2	6.83	152.7 (− 1.8)	158.7 (− 0.8)	0.5	2.6	5 / −	9.6	21	8.9	2.2	NA	−
12	F	−	7.5	7.7	126.1 (0.1)	15.9 (0.1)	2	8.83	153.6 (− 1.6)	160.5 (− 0.4)	0.2	4.2	13.2 / −	6.6	8	9.0	3.3	156.5 (− 1.1)	− 0.7
13	F	1° cousins	7.5	8.4	130.5 (0.3)	19.9 (1.4)	2	10.5	147 (− 2.7)	158.5 (− 0.8)	0.7	1.3	50.3 / −	12.4	10	8.7	2.3	NA	−
14	F	−	7.2	7.3	124.4 (0.4)	14.4 (− 0.8)	2	NA	NA	159.7 (− 0.6)	0.7	3.8	35 / −	10	12	−	−	159 (− 0.7)	− 0.1
15	F	−	7.1	7.1	126.6 (0.9)	18.1 (1.1)	2	8.83	154 (− 1.5)	154.2 (− 1.5)	0.1	2.4	7.6 / −	5	20.1	7.2	4.9	161 (− 0.4)	+ 1.1
16	F	−	7.6	7.6	117 (− 0.6)	16.1 (0.3)	2	7.83	149.4 (− 2.3)	155 (− 1.4)	0,1	1.9	5 / −	5	12	8.9	3.0	152.7 (− 1.8)	− 0.4
17	F	−	7.3	7.3	121.1 (− 0.4)	19.3 (1.5)	2	8.83	147.1 (− 2.7)	154.5 (− 1.5)	0.1	1.9	5 / −	6.2	15.4	8.2	3.0	NA	−
18***	F	−	6.5	7.2	124 (0.3)	16.3 (0.3)	4	7.83	158.5 (− 0.8)	158 (− 0.9)	0.2	7.8	2.6 / −	15.1	11	8.4	3.2	161 (− 0.4)	+ 0.5
Median IQR	−	−	7.3 (0.5)	7.4 (1.6)	130.5 (14.7) 0.3 (0.8)	17.3 (3.4) 0.7 (1.2)	−	8.83 (1.2)	156 (7.3)	159.4 (4.5) − 0.7 (0.6)	0.6 (0.5)	1.3 (2.8)	9.5 (12.3)/0.3 (0.1)	7.3 (6.6)	13.0 (5.5)	8.8 (0.7)	2.4 (0.8)	160 (5.2) 0.5 (0.2)	−

*F* female, *M* male, *yrs* years, *Cons* consanguinity, *SDS* standard deviation score, *BMI* body mass index, *PAH* predicted adult height, *LH* luteinizing hormone, *FSH* follicle stimulating hormone, *E2* estradiol, *T* testosterone, *GnRH* gonadotropin releasing hormone, *NA* not available

^+^(P.7) did not want to continue treatment

*DLK1 c.67+78C>T

**DLK1 c.357C>G/p.(Tyr119Ter)

***MKRN3 c.982C>T/p.(Arg328Cys)

^#^In certain instances, signs of puberty had already commenced before admission to the pediatric endocrinology outpatient clinic. In these cases, the onset ages of puberty were determined by the initiation of complaints. Consequently, in these cases, the age of referral is much higher than the onset time of CPP

### Molecular genetic results

**Fig. 1:**
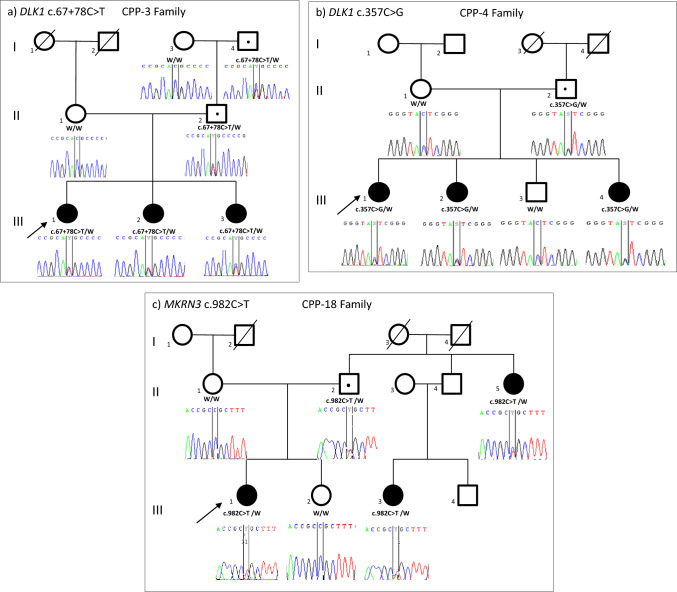
Pedigrees and sequencing data of three families **a** CPP-3 (III.1), **b** CPP-4 (III.1), **c** CPP-18 (III.1)) identified with the *MKRN3* and the *DLK1* gene variants. Arrow indicates probands, the open square indicates; male, and the open circle indicates; female. The (/) symbols represent deceased family members. The black symbols represent affected individuals, and the black dots represent asymptomatic carriers according to the imprinted patterns of inheritance. Mothers (**a**—I.3 and II.1, **b**—II.1 and **c**—II.1) without the mutation exhibited normal pubertal timing

Three variants in two genes were identified (Table 2); CPP-3 carried novel heterozygous c.67+78C>T/p.(?) in the first intron of the *DLK1* gene, CPP-4 disclosed a heterozygous variant, resulting a nonsense alteration, c.357C>G/p.(Tyr119Ter) in the *DLK1* gene, and CPP-18 had a previously reported missense alteration, c.982C>T/p.(Arg328Cys, rs1264639964), in the *MKRN3* gene (Fig. 1). The segregation analysis of the *DLK1* and *MKRN3* variants in the family demonstrated compliance with the paternal inheritance model. No pathogenic variants were detected in the *KISS1*, *KISS1R*, and *PROKR2* genes in individuals in the cohort.

**Table 2:** Molecular results of individuals with central precocious puberty carrying novel and known variants. ClinVar submission numbers of the novel variants shown in bold letters

Patients	Gene transcript protein	Genomic location (GRCh37)	Nucleotide	Peptide	Location type	Zygosity	MAF GnomAD	dbSNP/Clinvar	Varsome (ACMG classification)	Franklin (ACMG classification)	MT	Polyphen-2 (hum var)	Splice AI
CPP-3	*DLK1* NM_003836.7 NP_003827.3	chr14:101193550 C>T	c.67+78C>T	p.(?)	Intron 1 Splice site?	Heterozygous	NA	–/SCV003935250	Likely benign PM2, BP4	VUS PM2, BP7	Disease-causing	NA	0.45 > 0.43 (DL score)
CPP-4		chr14: 101198473 C>G	c.357C>G	p.(Y119*)	Exon 4 nonsense			–/SCV003935249	Likely pathogenic PVS1, PM2	VUS PM2			NA
CPP-18	*MKRN3* NM_005664.3 NP_005655.1	chr15: 23811911 C>T	c.982C>T	p.(R328C)	Exon 1 missense		< 0.00001	​rs1264639964 VCV000438339.1	Likely Pathogenic PP3, PM1, PM2, PP5	VUS PM2, PP5		Probably damaging (Score: 1)	NA

*MAF* minor allele frequency, *Het* heterozygous, *VUS* variant of uncertain significance, *PM1/PM2* pathogenic supporting, *BP4* benign moderate, *BP7* benign supporting, *PVS1* pathogenic very strong, *PP3/PP5* pathogenic strong, *NR* not reported, *NA* not applicable, *DL* donor loss, *MT* mutation taster

### Measured serum DLK1 levels

The range of serum DLK1 levels in the control group was from 411.1 to 1125.5 ng/L. The mean serum level in the control group was 680.9 ng/L. CPP-3 family DLK1 serum results (ng/L): III.1: 260.2, III.2: 207.0, III.3:242.4, II.1:510.5, III.2: 378.1

**Fig. 2:**
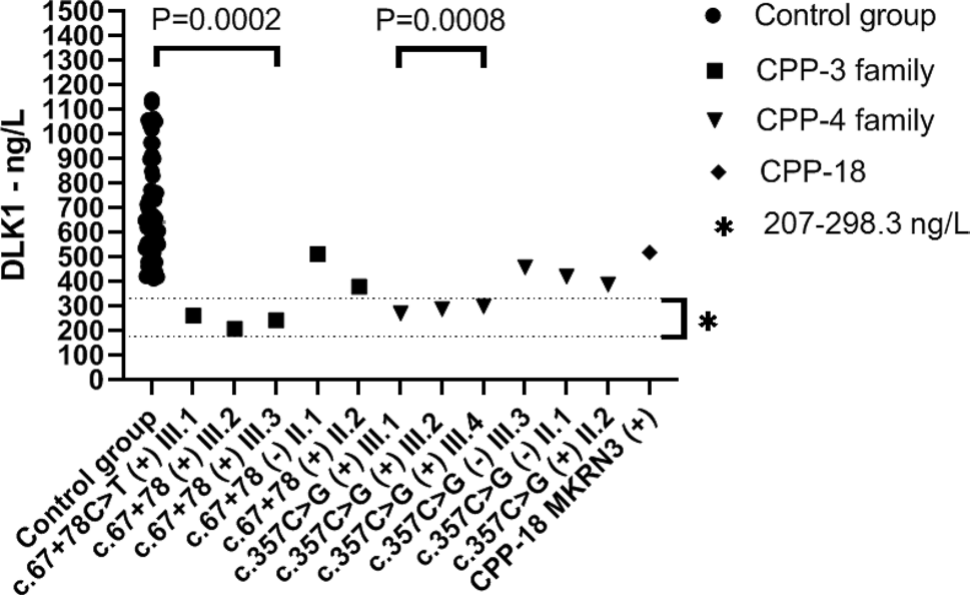
DLK1 serum levels in affected family members (CPP-3, CPP-4), CPP-18 case, and control groups. Affecteds had low detectable DLK1 serum levels compared to the control group. The symbol * represents the range of the lowest and highest serum levels detected in affected individuals. The assay limit of sensitivity of ~ 10 ng/L

CPP-4 family DLK1 serum results (ng/L): III.1: 269.2, III.2: 287.1, III.3:457.1, III.4: 298.3, II.1: 419.3, III.2: 386.6. and CPP-18: 518.1 (ng/L). The serum DLK1 levels in the affected CPP-3 family members carrying the c.67+78 variant mean of DLK1 serum 236.6 ng/L, while in the CPP-4 with the c.357C>G variants mean 285 ng/L. Additionally, the serum levels of DLK1 were found low in affected individuals of these families carrying the variant in the paternally derived allele (CPP-3; III.1, III.2 and III.3, and CPP-4; III.1, III.2 and III.4) versus the control group. The difference was statistically significant in both serum level evaluations (*p* < 0.05). Measurable serum DLK1 levels were detected in all patients and controls in whom serum DLK1 levels were examined. This is because the test can detect serum DLK1 levels equal to or higher than approximately 10 ng/L. However, a significant difference was detected in affected individuals in CPP-3 and CPP-4 families compared to the control group (*p* < 0.05) (Fig. 2). Interestingly, unaffected fathers in both families had lower serum levels compared to the control group, but the fathers' serum results were not statistically significant compared to the control group (*p* = 0.105). In addition, serum DLK1 level was also examined in CPP-18 with MKRN3 pathogenic variant, but no statistically significant difference was revealed (*p* = 0.403).

## Discussion

In this study, the genetic etiology in 18 cases with a family history of early puberty with respect to variants in 5 genes associated with the CPP were investigated. One pathogenic variant in the *MKRN3* and two novels in the *DLK1* gene were found compatible with maternal imprinting in family segregation analysis. Low serum DLK1 levels in two individuals supported the association of the novel alteration in the *DLK1* gene with the clinical findings. Currently, 64 inactivating variants have been described in the *MKRN3* gene associated with CPP [1, 6, 11, 30, 31]. A meta-analysis of CPP cases, encompassing 22 studies from 17 countries, determined that 89 (76 girls, 8.6%, and 13 boys, 1.4%) of 880 CPP (including sporadic and familial forms) cases had been found with variants in the *MKRN3* gene [30]. Studies from Türkiye have shown the association of *MKRN3* only in the familial CPP cases. In their study, Şimsek et al. [32] reported two different frameshift mutations in the *MKRN3* gene in two females from two unrelated families that underwent exome sequencing (WES). The first of these mutations was heterozygous c.802_803del/p.(Met268Valfs*23), while the second was heterozygous c.441delG /p.(His148Thrfs*23). Both alterations had been paternally inherited. In large segregation, variants had been detected in seven affected members and 11 unaffected family members. In a recent study, using the WES technique, Aycan et al. [33] analyzed 19 individuals (17 females and 2 males) of CPP from 10 families and reported a heterozygous c.630_650delinsGCTGGGC/p.(Pro211Leufs*16) variation causing frameshift in one male patient (1/19, 5.3%) at Tanner stage II (testicular volume > 4 mL). The pathogenic effect of the variant was supported by in silico analyses and its segregation was consistent with a paternal inheritance model. In another study conducted by Kırkgöz et al., a total of 102 patients with CPP were included, of which 53 had a family history of CPP. In the study, novel variants were identified in two (3.8%) patients; one with a family history of CPP and one (2%) without a family history. The identified variants included a novel heterozygous c.1A>G (p.Met1Val) alteration, a novel heterozygous c.683_684delCA/p.(Ser228*), and a previously reported c.482dupC/p.(Ala162Glyfs*15) frameshift variation. Overall, potentially pathogenic variants in the *MKRN3* gene were detected in 2.9% of the total cohort and 3.8% of the cases with a family history of CPP [34].

**Fig. 3:**
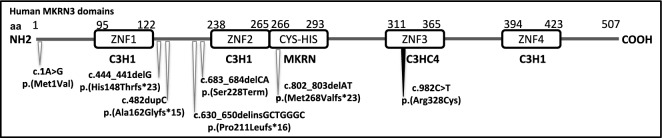
Schematic structure of the MKRN3 protein (507 aa/Q13064-Uniprot). The variants were reported from Türkiye (white arrow) and disclosed in this study (black arrow). aa; amino acids, ZNF; Zinc-Finger domains, Cys-His; Makorin type zing finger domain

Despite the CPP-associated MKRN3 gene variants have been reported more frequently in the literature, a variant was detected in only one case (CPP-18, 5.5%) in our study. The c.982C>T/p.(Arg328Cys) variant, which has been shown to be loss-of-function in previous investigations, is known to be frequently associated with CPP in the *MKRN3* gene. In previous studies, the first pubertal signs in subjects with the c.982C>T variant were reported between 6 and 7 years of age [35, 36]. The case (CPP-18) with the c.982C>T variant presented with Tanner stage IV at the age of seven years. While it has been reported that subjects carrying this variant may exhibit FSH dominance in GnRH stimulation tests, our case clearly showed LH dominance. The family segregation analysis revealed compatibility with the paternal inheritance, as found in the patient’s father, paternal aunt, and paternal cousin. The *MKRN3* variants reported in cases from Türkiye [32–34], and shown in our study are presented in Fig. 3. Since the identification of pathogenic variants in *MKRN*3, several research groups have reported their findings. There is comparatively less data available regarding variants in *DLK1* in the context of CPP.

The association with the *DLK1* gene with CPP was first reported in four sisters (two sisters and two paternal half sisters) carrying a paternally inherited deletion of approximately 14 kilobases, including the 5' untranslated region and exon 1 and duplication of 269 bp in intron 3 of the *DLK1* gene. These four siblings presented with advanced pubertal signs at very young ages (thelarche between 4.6 and 5.9 years, median 5.2 years) and laboratory tests clearly supported the presence of puberty. Besides the absence of detectable circulating DLK1 levels clarified the impact of the genomic deletion on DLK1 production in these siblings [10]. In the current study, we detected two distinct *DLK1* variants in two different families (Fig. 1a, b). The onset of puberty signals in the affected sisters from both families occurred between 5.7 and 7.5 years of ages (median 6.6 and 7.3 years, respectively). The findings of the sisters of the patients with *DLK1* variations are shown in Table 3.

**Table 3:** Clinical and hormonal findings of affected individuals with DLK1 variants

Member	Sex	Cons	At diagnosis													The age of the start of GnRH treatment (yrs)	The duration of GnRH treatment (yrs)	Final height cm (SDS)	ΔFinal height-target height (SDS)
			The age of puberty onset (yrs)	The age at the referral time (yrs)	Height cm (SDS)	BMI kg/m^2^ (SDS)	Puberty tanner	Bone age (yrs)	PAH cm (SDS)	Target height cm (SDS)	LH (IU/L)	FSH (IU/L)	E2 (pg/mL)	Stimulated LH (IU/L)	Stimulated FSH (IU/L)				
3* (III.1)	F	Siblings	7	7.4	136.3 (2.5)	19.2 (1.4)	4	8.83	166 (0.5)	160 (− 0.5)	0.6	1.4	15	3.3	12.8	7.4	3.6	167.4 (0.84)	+1.34
CPP-3 III.2*			7	8.8	145.3 (2.1)	15.1 (1.2)	3	10	163.3 (0.3)	160.1 (− 0.5)	0.6	1.5	5	5.1	9.1	9.1	2.5	164.6 (1.0)	+ 1.5
CPP-3 III.3*			5.7	5.7	112.1 (− 0.3)	16.7 (0.7)	2	5	NA	159.5 (− 0.6)	0.3	1.3	5	5.2	9	−	−	NA	−
4** (III.1)		Siblings	7	8.4	138.9 (1.8)	16.3 (0.1)	4	10.5	155.5 (− 1.3)	162.3 (− 0.1)	1.6	6.6	76	−	−	8.7	2.1	156.8 (− 1.1)	− 1.0
CPP-4 III.2**			7.5	8.9	145.3 (2.4)	15.11 (− 0.62)	3	12	161.3 (− 0.3)	164.5 (0.24)	5.2	5	102	−	−	−	−	NA	−
CPP-4 III.4**			7.1	7.7	118.4 (− 1.2)	11.7 (− 0.7)	2	9	150 (− 2.2)	162.3 (− 0.1)	2.5	3.3	37.6	−	−	7.9	√	NA	−

*F* female, *M* male, *yrs* years, *SDS* standard deviation score, *BMI* body mass index, *PAH* predicted adult height, *LH* uteinizing hormone, *FSH* follicle stimulating hormone, *E2* estradiol, *GnRH* gonadotropin releasing hormone, *NA* not available, *Cons*. consanguinity, *√* treatment still continue

*DLK1 c.67+78C>T

**DLK1 c.357C>G/p.(Tyr119Ter)

In further studies, four additional loss-of-function *DLK1* variants (c.401_404+8del/p.(?); p.(Gly199Alafs*11); p.(Val271Cysfs*14) and p.(Pro160Leufs*50)) have been reported [17, 37]. In the first of these studies, researchers investigated a group of 60 unrelated children or adult patients who had a history of CPP or early menarche, and they did not have any mutations in the *MKRN3* gene. They revealed that *DLK1* gene mutations were found in five women from three unrelated families (two affected sisters in family 1, index women in family 2, index girl and her paternal aunt in family 3). In this group, thelarche was reported between 4.6 and 7 years (median: 5 years) and menarche between 7 and 9 years in untreated women. It is also reported that serum DLK1 concentrations were not detected in three affected women from two families (family 1/ p.(Gly199Alafs*11) and family 3/ p.(Pro160Leufs*50)) with frameshift variants detected, selected for serum level investigation. [17].

In the second study, a rare variant at the splice site junction of *DLK1* (c.401_404+8del) was reported in a girl with CPP with the onset of pubertal signs at 5.7 years of age and first clinical evaluation at 6.3 years (Tanner stage III). Family segregation analysis showed that the *DLK1* deletion was de novo in the affected child. Serum DLK1 levels were not detected in this case (< 0.4 ng/mL)[37].

**Fig. 4:**
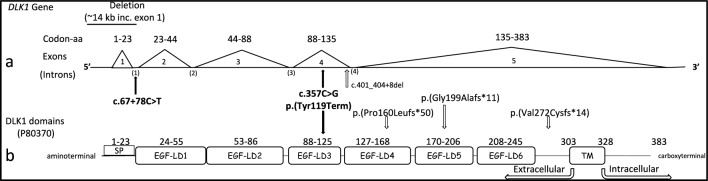
Schematic representation of the *DLK1* gene (**a**) and DLK1 peptide (P80370-Uniprot) (**b**). The protein structure has one signal peptide (1–23aa), six tandem epidermal growth factor (EGF)-like repeat domains (24–245 aa), an extracellular region (246–303aa), a transmembrane region (304–327aa), and a cytoplasmic region (328–383aa). *TM* transmembrane, *aa* amino acids, *EGF-LD* EGF-like extracellular domains. The DLK1 gene variants previously reported in the literature are shown in nucleotides and peptides. Novel variants revealed in this study are written in bold letters

In contrast to the ages at which puberty signals started in the cases with deletion and frameshift mutations detected in the literature, CPP-3 and CPP-4 cases with intronic variant detected in the DLK1 gene in our study were 7 years old when puberty signals started, while Tanner stages were stage IV [10, 17, 37]. In studies conducted to investigate the effect of variants on DLK1 production in individuals with deletion or frameshift mutations, serum DLK1 levels were measured and serum DLK1 levels were reported to be undetectable. This suggests that the deletion or frameshift mutation leads to a complete lack of DLK1 production or has affected DLK1 expression in these individuals [10, 17, 37]. In our study, serum DLK1 was measured by ELISA assay to investigate the effect of nonsense and intronic variants on DLK1 production, which has not been previously reported in the literature. In previous studies, deletion or frameshift mutations have been reported, and in those cases, serum levels were not detected. In our study, on the contrary, low DLK1 levels were detected. This was thought to be due to the high sensitivity of the test used in our study and the fact that the reported mutation types were different from those in the literature. In the present study, the novel intronic c.67+78C>T and exonic c.357C>G variations disclosed in CPP-3 and CPP-4 showed consistent inheritance patterns (Fig. 4).

Previous studies have indicated that the nonsense mutations resulting in the conversion to stop codons in the transcript can lead to premature translation termination and the production of shortened polypeptide products. It is anticipated that such mutations may either result in the formation of a truncated peptide, or nonsense mRNA decay of the transcript, which is likely to have a deleterious effect [39]. Furthermore, low serum DLK1 levels of the cases carrying c.67+78C>T and c.357C>G supported the causative nature associated with CPP in our patients. Deep learning splicing prediction analysis (SpliceAI) of c.67+78C>T striking the pre-mRNA of the *DLK1* gene showed a mild change of the score from 0.45 to 0.43. On the other hand, the Multiz Alignment Configuration analysis (PhyloP470) of the alteration over default mammalian species (University of California Santa Cruz Genome Browser) showed that “C” is highly conserved. The “mean” of PhyloP470 score for pathogenicity is reported as 3.921 and for “benign” as − 0.194 for the non-coding regions [38]. The PhyloP470 score of c.67+78C>T was 2.377. Therefore, the highly conserved nature of c.67+78C>T and the loss-of-function prediction of the c.357C>G/p.(Tyr119Ter), both suggested the pathogenic nature of those variants in the *DLK1* gene. The DLK1 nonsense variant detected in this study is predicted to affect the linkage on (EGF)-like repeat domains 3, encoding by exon 4 [17], leading to protein truncation.

*DLK1* is a paternally expressed gene; affected individuals inherit mutations from their fathers. In the segregation analysis of the CPP-3 family, the father (III.2, age 38, height 172 cm, weight 84 kg) inherited the variant from his father (I.4, age 65, height 169 cm, weight 71 kg) following the expected inheritance pattern. Surprisingly, despite this, father (III.2) reported no clinical findings or complaints related to CPP. He could not recall the age of his pubertal development. This situation is presumed to stem from the fact that paternal inheritance is often insufficiently recognized in men or that testicular enlargement is not very noticeable compared to breast development and menarche in girls [14].

Since gain-of-function alterations in the *KISS1* and *KISS1R* genes were understood to be the genetic cause of CPP, the association of these genes with sporadic cases has been reported very rarely [7, 8]. Similarly, a mutation in the *PROKR2* gene has only been shown in one case so far [9]. In our study, no pathogenic variants were detected in these genes, consistent with the literature.

## Conclusion

Our study showed that the diagnostic utility of sequencing the *DLK1* and *MKRN3* genes contributed 16% to the genetic etiology of CPP, which provides supportive information when genetic counseling to families.

Although the clinical findings of the patients are followed up in terms of endocrine discipline, knowledge of the genetic diagnosis is a guide for genetic counseling to be given to patients and may contribute to clinical follow-up and treatment plan. Our study also revealed that causative CPP genes might be associated with a minor proportion of the development of CPP. It may be expected that next-generation sequencing of the whole genome may support discovering newly associated genes or novel variants, especially in the conserved regulatory or deep intronic regions.

## Data Availability

All data generated or analyzed during this study are included in this article. Further inquiries can be directed to the corresponding author.
